# Proteomics Applications in Health: Biomarker and Drug Discovery and Food Industry

**Published:** 2018

**Authors:** Nasrin Amiri-Dashatan, Mehdi Koushki, Hojjat-Allah Abbaszadeh, Mohammad Rostami-Nejad, Mostafa Rezaei-Tavirani

**Affiliations:** a *Proteomics Research Center, Shahid Beheshti University of Medical Sciences, Tehran, Iran. *; b *Department of Biochemistry, Medicine Faculty, Tehran University of Medical Sciences, Tehran, Iran. *; c *Hearing Disorders Research Center.Shahid Beheshti University of Medical Sciences, Tehran, Iran. *; d *Research Institute for Gastroenterology and Liver Diseases, Gastroenterology and Liver Diseases Research Center, Shahid Beheshti University of Medical Sciences, Tehran, Iran.*

**Keywords:** Proteomics, Proteome profiling, Foodomics, Biomarker, Drug discovery

## Abstract

Advancing in genome sequencing has greatly propelled the understanding of the living world; however, it is insufficient for full description of a biological system. Focusing on proteomics has emerged as another large-scale platform for improving the understanding of biology. Proteomic experiments can be used for different aspects of clinical and health sciences such as food technology, biomarker discovery and drug target identification. Since proteins are main constituents of foods, proteomic technology can monitor and characterize protein content of foods and their change during production process. The proteomic biomarker discovery is advanced in various diseases such as cancer, cardiovascular diseases, AIDS, and renal diseases which provide non-invasive methods by the use of body fluids such as urine and serum. Proteomics is also used in drug target identification using different approaches such as chemical proteomics and protein interaction networks. The development and application of proteomics has increased tremendously over the last decade. Advances in proteomics methods offer many promising new directions of studying in clinical fields. In this regard, we want to discuss proteomics technology application in food investigations, drug, and biomarker discovery.

## Introduction

 Proteins are key players in cellular function and the proteomics technology is a powerful tool for the study of total expressed proteins in an organism or cell type at a particular time. Since proteins are responsible for the function of the cells, after completion of genome sequencing, most of the biological questions remained unanswered. It is known that expression, localization and activity of proteins differ in various conditions, therefore study of protein expression in cell types or different conditions will be important for identifying and understanding of their biological information. Proteomic analysis can provide comprehensive assessment of cellular activities in clinical research of different diseases. The proteome term was used for the first time in 1994- and proteomics is the main tool for proteome research. Proteomics methods have several applications in various fields especially in health science and clinics. On the other hand, proteomics methodologies are being used in the food science. Human food safety and its quality control is a very essential process. In this regard, food-proteomics plays important roles in the identification of protein composition during the food production. The terminology of industrial proteomics used frequently in last years (1), that this method is used for validation and control of food products (2). Identification of drug targets in the drug and biomarker discovery pipeline to diagnose and fight human disease is the first and key step. This step is critical and important because a drug target must satisfy a variety of criteria. Since proteins and interaction between them are fundamental in biological systems, proteomics can also be a valuable approach for drug target discovery. In this mini review, proteomics applications in the field of food industry, drug and biomarker discovery are presented.

*Proteomics Application in Biomarker discovery*

**Figure 1:**
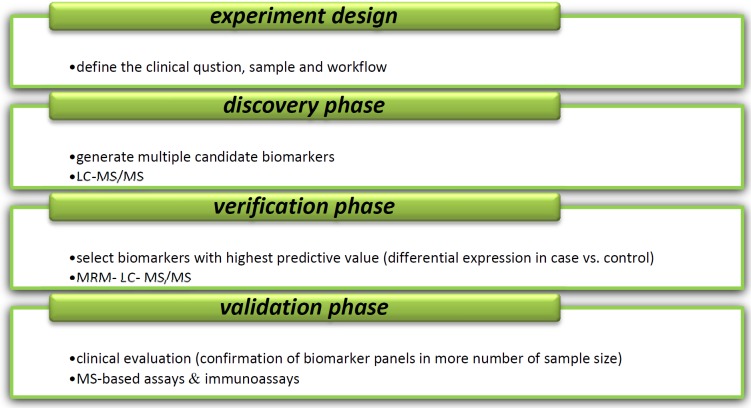
Different steps of new biomarker development. Majority of methods for analysis of disease- specific biomarkers are based on mass spectrometry (MS). Variety of separation methods including liquid chromatography (LC), electrophoresis (E), two-dimensional electrophoresis-MS (2DE-MS), 2D-polyacrilamid gel electrophoresis-MS (2D-PAGE-MS), matrix- assisted laser desorption/ionization- time of flight-MS (MALDI-TOF-MS), surface- enhanced laser desorption/ionization-TOF-MS (SELDI-TOF-MS), LC-MS/MS, Fourier transform ion cyclotron resonance- MS (FTICR-MS), multiple reaction monitoring/ selected reaction monitoring (MRM/SRM) in combination with MS use in discovery step of biomarker identification process. In validation step, several techniques such as enzyme- linked immunosorbent assay (ELISA), arrays, MRM/SRM, western blot (WB) and immune histochemistry (IHC) can be used (91, 92).

 A biomarker usually refers to disease- related proteins or a biochemical indicator that can be used in the clinic to diagnose or monitor the activity of disease, prognosis, and development of the disease, and also to guide the molecular target treatment or evaluation of the therapeutic response (3, 4). In medicine, a biomarker can be a traceable substance that is introduced into an organism as a means to examine organ function or other aspects of health. One example of a commonly used biomarker in medicine is PSA (prostate specific antigen). Nevertheless, malignancies are usually detected at severe stages when patients have very poor prognosis and few treatment options are present which are mostly due to a high cost and time -consuming process in biomarker tracing. Furthermore, development of a better throughput analyzing method is a critical requirement for early detection; and combination of various platforms of onco-proteome data is required (Figure 1) (5). As protein expression alters during disease condition in biological pathways, monitoring of these altered proteins in tissue, blood, urine, or other biological samples can provide indicators for the disease (6). Proteomics technology has been extensively used in the molecular medicine especially for biomarker discovery. By analyzing of a global protein profiling in the body fluids, proteomics can identify invaluable disease-specific biomarkers. Expression of proteomics provides biomarker detection through comparison of protein expression profile between normal samples vs. disease affected ones. The simplest approach used in biomarker discovery is 2D-PAGE in which protein profiles are compared between normal and disease samples such as tumor tissues and body fluids (7). Disease-specific biomarkers can be divided into diagnostic, prognostic, and treatment predictive biomarkers according to information which they provide (8). A diagnostic biomarker is used for early detection or presence of the disease. A prognostic biomarker usually is used to predict the recurrence and aggression of disease and a patient response to treatment by a given drug. Predictive biomarkers are useful tools to classify the patients into responder and non-responder groups (7). This classification also is important in drug design applications (9). According to estimates, only 2% of human diseases appear to be due to a single gene damage, and epigenetic and environmental factors involved in the development and outcome of the diseases account for remaining 98% (10). In this regard, proteomics can be helpful to identification of proteins that can potentially serve as disease-associated biomarkers which involved in disease progression. After identifying biomarkers by mass spectrometry-based approach, biomarkers need to process using bioinformatics analyses and also need to be reproduced in different populations (11). Despite amplifying the interest, high investigation burdens, and high level of publications, unfortunately, a few of identified biomarkers using proteomics technology have been validated and approved by FDA for clinical usage yet (12, 13).

**Figure 2:**
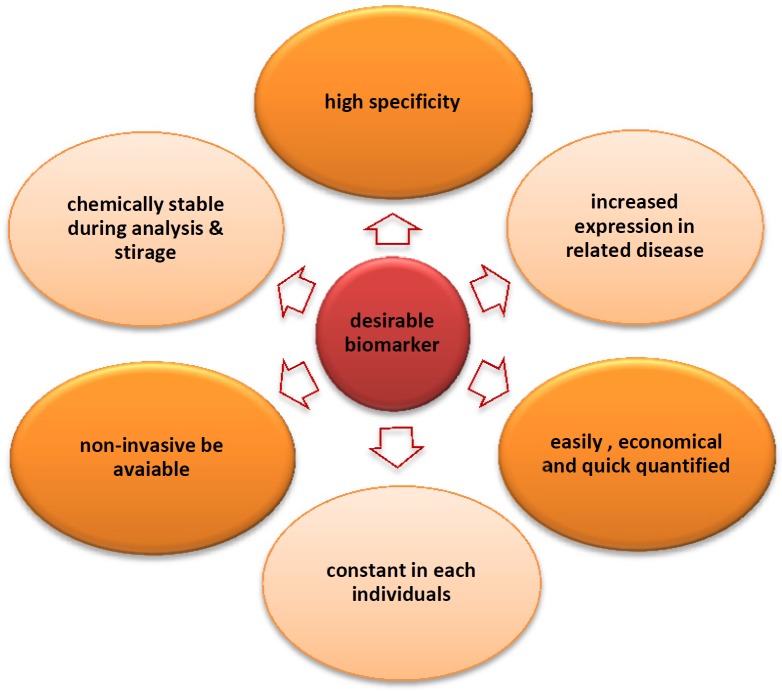
Ideal biomarkers features. The ideal biomarker should have high specificity for a certain disease condition. Proteomics technology is powerful tool for biomarker discovery through characterization and evaluation of global profiling of proteins under given state

 Concerning biomarker discovery, proteomics technology is a promising tool for disease-associated biomarker detection in the biological fluids including urine, plasma, serum, etc. (Table 1). It is very important that body fluid samplings for proteomics research are less invasive and have low-cost advantages. The proteomics biomarker discovery is advanced in a variety of diseases such as cancer (14-16), cardiovascular diseases, acquired immune deficiency syndrome (AIDS), renal diseases, diabetes (17), etc. However, despite many technological developments, some challenges remain to be overcome as body fluids are highly complex mixtures of proteins and contain high dynamic range of proteins. Each of these sample types can be used in different disease conditions, for example in kidney disease; urine is a valuable sample because of urine proteins directly reflect changes in the kidney function (18). Blood is a logical fluid to be used for biomarker discovery in human disease, since it has several merits over other samples presented in Figure 2. On the other hand, biomarker identification using plasma proteomics has challenges including (i) high dynamic range of plasma proteins, (ii) low abundance of invaluable biomarkers in plasma, and (iii) patients’ variation (19). Despite the advancement of proteomic methods, the current single method cannot simultaneously conquer the above challenges in biomarker discovery process in plasma proteome yet. 2D-PAGE/MALDI-TOF and surface-enhanced laser desorption/ionization (SELDI)/Protein Chip techniques are the most approaches used to identify biomarkers for different disease (6). SELDI-MS is used for biomarker detection in a variety of diseases due to its ease of operation and high-throughput application (20).

**Table1:** Proteomics-based biomarker discovery in different biological fluids such as blood (plasma or serum), CSF, urine, saliva, CSF and tissue/ or cells

**Sample/ Disease**	**Method**	**Potential biomarker**	**Study**
Serum DS Epilepsia	2D-DIGE, 2D-CF, MudPIT; LC/LC-MS/MS, MALDI-TOF-MS MS, ELISA	Alpha-1-acid, glycoprotein SAA	Nagalla et al., 2007 (63) Li et al., 2013 (64)
Plasma PD Neuroblastoma AD Gastric cancer	iTRAQ, MALDI-TOF-TOF, MRM, LC-MS/MS LC-MS/MS iTRAQ-MS, ELISA 2DE-MS, WB	Tyrosine-kinase, non-receptor-type 13, Netrin G1 Complement C3 Complement 4a CFI precursor	Lehnert et al., 2012 (65) Kim et al. 2014 (66) Bennett et al., 2012 (67) Liu et al., 2007 (68)
Urine ADPKD Bladder cancer FSGS	CE-MS Shotgun proteomics, ELISA nLC-MS/MS	PKD1, PKD2 Midkine, HA-1 77 putative biomarker such as: CD59, CD44, IBP7, Robo4, and DPEP1	Kistler et al., 2009, 2013 (69, 70) Shimwell al., 2014 (71)
Saliva Diabetes type2 OSCC Gastric cancer Oral Cancer GCF Periodontal disease	2D-LC-MS/MS, WB SDS-PAGE, LC-MS/MS tandem mass tags (TMT) shotgun proteomics LC-MS/MS	G3P, SAA, PLUNC, TREE resistin (RETN) CSTB, TPI1, DMBT1 M2BP, MRP14, CD59, Profilin, Catalase Moesin	Border et al., 2012 (72) Wu et al., 2015 (73) Xiao et al., 2016 (74) Hu et al., 2008 (75) Tsuchida et al., 2014 (76)
CSF AD Multiple sclerosis	Nano-LC-MRM/MS, ELISA 2DE-MS	24 peptides CRTAC-1B,tetranectin	Choi et al., 2013 (77) Hammack et al.,2004 (78)
Tissue Breast cancer Colon Cancer BCC cell culture media REH cell line	iTRAQ, SRM/MRM, LC-MS/MS, WB, IHC Label-free LC-MS/MS, WB, IHC 2DE-MS- MALDI 2DE-MALDI-TOF/TOF-MS	GP2, MFAP4 RAI3 C3b,aldolase C, FGG, Prx-cis, Protrombin, VDAC and LRG VDAC1, SNX3, PFDN6	Muraoka et al., 2012 (79) Zougman et al., 2013 (80) Zali et al., 2010 (81) Dehghan-Nayeri et al., 2016 (82)

AD (Alzheimer disease), PD (parkinson disease), ADPKD (autosomal dominant polycystic kidney disease), OSCC (oral squamous cell carcinoma), CSTB (cystatin B), TPI1 (triosephosphate isomerase), DMBT1 (deleted in malignant brain tumors 1 protein). FSGS (Focal Segmental Glomerulosclerosis)**, **BCC (Basal Cell Carcinoma), VDAC1 (voltage dependent anion channel 1), SNX3 (sorting Nexin 3), PFDN6 (prefoldin subunit 6), GCF (Gingival Crevicular Fluid).

 Due to cancer importance, the most proteomics studies in the field of biomarker discovery concerned cancer diseases. Proteomics as one of the modern areas of biochemistry holds great promise in the cancer study. Since proteome represents actual state of the cell, tissue, or organism, there are suitable biomarkers related to the tumors which can be used for diagnostic proposes or follow up of patients (5). It should be noted that one of the major challenges facing the biomarker discovery especially in cancer is heterogeneity between patients (7). Therefore, personal medicine has become a popular trend for cancers. The combined use of biomarkers as biomarker panels has also been shown to give a better prognosis and increase sensitivity and specificity to predict the response of patients to therapy (21). Since advantages of biomarker panels (or biomarker patterns) have been confirmed in several publications recently, in this context proteomics can be useful tool for identification and verification of such biomarkers and panels (19).

*Application of Proteomics in Drug Discovery*

**Figure 3:**
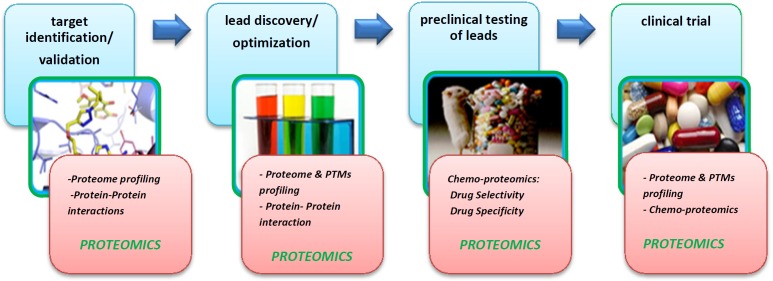
Drug discovery process. Proteomics technology by protein analysis (global protein profiling, protein- protein interaction profiling, PTMs profiling and chemo-proteomics) contributes in different steps of drug discovery process that shown with red color

 Over the past few years, proteomics study based on MS has expanded its role in almost all diverse research fields of science. As the drug discovery is an inherently complex process and values high-cost, new emerging technologies such as proteomics can facilitate and accelerate discovery processes. It is estimated that finding each new drug candidate costs $70 million (22). Drug discovery has many stages which have been presented in Figure 3 and indeed it is a multidisciplinary field using genomics, proteomics, metabolomics, bioinformatics, and system biology. As is clear in Figure 3, proteomics plays a major role in target identification step. Proteomics studies also are useful for drug action, toxicity, resistance, and its efficacy under examination. Although genomics is well applicable in the drug discovery process, yet proteomics-based methods are more advantageous than genomics for the following reasons: (i) Gene expression profile is not indicative of protein expression (23) and (ii) Genomics is capable of presenting cellular events only to the transcriptome stage (24). Therefore, most investigations focus on proteomics studies which evaluate proteins and their post-translational modifications (PTMs) in large-scale. Target identification is the first step in drug discovery process and the identification and early validation of disease-modifying targets are essential steps in the drug discovery pipeline. Indeed, proteomics technology plays a prominent role in the target proteins identification, as proteins are main drug targets in disease conditions (25). Proteomics refers to the cell protein profile analyses. It is typically associated with system biology which is able to select protein biomarkers. Also in drug discovery pipeline, comprehensive understanding of disease-associated pathways plays an important role in order to design a compound to inhibit or augment a certain chemical pathway or cycle (26). This micro-molecules (drugs) act on protein targets, which usually are in interactions with other proteins in a cellular network. In order to understand this network of protein interactions, the use of proteomics-based technology is reasonable to discover the drug candidates on their protein targets and revealing of cellular mechanisms resulting in the observed phenotype (27, 28). Recently, proteomics technology has made considerable progression in several platforms (29). Proteomics through several techniques , such as isotope coded affinity tags (ICAT) (30), stable isotopic labeling by amino acids in cell culture (SILAC) (31), isobaric tags for relative and absolute quantification (iTRAQ) (32), activity-based probes (ABPs) (33) and protein arrays (34), is utilized in multiple steps of drug development pipeline including target and lead identification (35). By global searching of the proteome in a given sample such as tissue or cells treated with a drug, proteomics provides insights about disease-related molecular and cellular mechanisms that facilities drug target discovery. Zali *et al*., 2015 evaluated protein targets of Lavandula angustifolia on the treatment of rat Alzheimer᾽s disease using 2D-PAGE-MALDI-TOF/TOF. In this study, some proteins presented as to be potential drug targets (25). 

**Figure 4:**
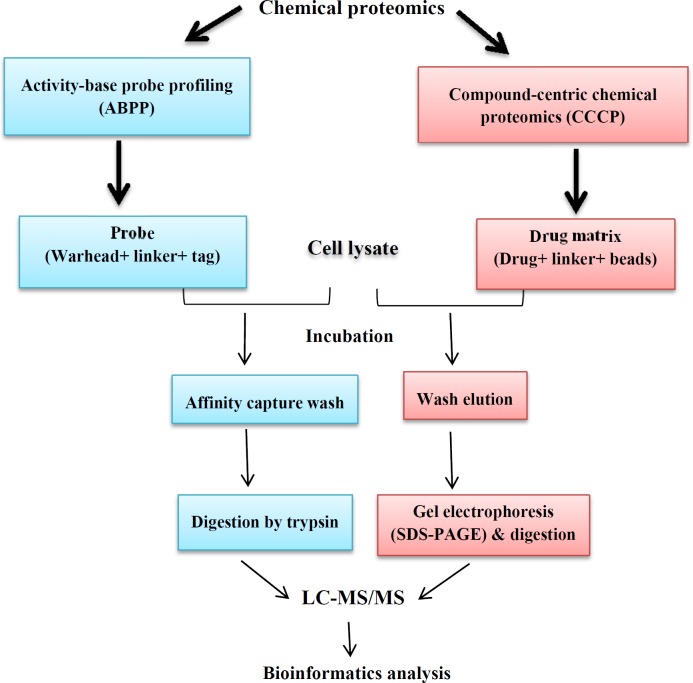
Comparison of activity-based probe profiling and compound-centric chemical proteomics

 The various MS-based platforms, including clinical, functional, and chemical proteomics are also involved in the modern drug discovery process. In the complex drug discovery pipeline, the various MS-based proteomic approaches extend beyond the common objective of drug target discovery, enabling the study of drug-target interaction (selectivity and specificity), drug activity (efficacy, resistance, toxicity) and elucidating the mechanism of action of a new drug (36, 37). Functional proteomics is a research discipline in proteomics-based field which pursues the two main objectives, including explanation of the biological function of unknown proteins and the definition of cellular mechanisms at the molecular level (38). These data may help to develop new drug target identification. Functional proteomics is applicable to preparation information about protein identification, abundance, function, activity, interactions and PTMs; all of these aspects help to functional understanding of biological systems (36). Functional proteomics have been used in many investigations for drug discovery in a variety of pathological conditions (39, 40). This approach often is used in order to achieve information about signaling pathway of proteins, disease pathogenesis, and interaction of drugs with proteins. These data provide information through characterization of special group of proteins in response to signals (6). A new research area in proteomics field has been named chemical proteomics. Chemical proteomics as MS-based affinity chromatography approach identifies interactions of small molecules and proteins in proteome-wide state (41). Chemical proteomics can be performed for functional annotation of previously uncharacterized proteins for any cell type and tissue used. Furthermore, chemical proteomics represents one of the most direct approaches to investigate drug action mechanism and toxicity of drugs in physiological and clinical conditions. This approach is also able to identify novel enzymatic proteins and has the potential to accelerate the discovery of new drug targets (42). Moellering and Cravatt in 2012 proposed that chemical proteomics method can help to drug target identification in drug discovery process (43). Chemical proteomics also contributes in protein-protein interactions identification using affinity purification followed by mass spectrometry. Chemical and protein interaction proteomics differ in their definitions. This means that in protein interaction proteomics, interactions among proteins in a complex are evaluated after protein complex purification of cells which contain valuable information for protein-protein interactions characterization. In chemical proteomics approach, the chemical bait- protein interaction *in-vitro* happens (41). Identified targets using chemical proteomics can be valid. In fact chemical proteomics help to screen the compounds library and identify all of the proteins that interact with this library. Also using this technique, the side effects of drugs can be assessed. For example, chemo-proteomics is used in clinical candidate targeting of heat shock protein _90_ (44). Overall, the recent advances in chemical proteomics have provided new drug discovery strategies. These methodologies are providing complementary approaches to drug screening, drug target identification, and selectivity profiling. There are two variants of chemical proteomics involved in drug discovery. (I) activity-based probe profiling (ABPP), which evaluates a certain protein family in terms of enzymatic activity, and (II) compound-centric approach, which characterizes molecular mechanism of action of bioactive micro-molecules (41) (Figure 4). In compound-centric approach, the given compounds chemically bond to the matrix. Then the formed drug-matrix incubates with the lysate of interest. The captured proteins are eluted and separated using electrophoresis methods (SDS-PAGE). Subsequent proteolysis digestion peptide mixture is produced and then evaluated with MS and bioinformatics analyses. Compound- centric variant of chemical proteomics also contributes for introducing new targets through identification of protein binders without enzymatic function (45). In the activity-based probe profiling approach, the probe molecule and protein are bound covalently together. The probe consists of a reactive ligand, a linker group, and a tag. During the experiment, ligand is bound to the target protein and also the tag is used for the target protein identification and purification. The linker region is positioned between the ligand and the tag (46). Clinical proteomics provides discovering and understanding the protein roles in various pathological conditions to facilitate and accelerate the early diagnosis, prognosis, and identification of novel therapeutic targets and also for assessment of treatment response (47, 48). So, the application of proteomics tools in the field of medicine may facilitate the understanding of disease pathogenesis and discovery of new drug targets (49). In summary, drug discovery is a complex and expensive process that various platforms of proteomics-based approaches including functional, chemical, and clinical proteomics are being used to identify new drug targets. In practice, this is not easy because an infinite number of genes, proteins and other molecules interact with each other in the signaling pathways to direct cell function (50). On the other hand with recent progresses in the proteomic methodologies, there is an increasing interest in the application of these novel technologies to improve the drug-discovery process. Because the majority of the drugs act on protein targets, it is important that drug-discovery efforts focus at this level.

*Application of Proteomics in Food Industry*

**Figure 5:**
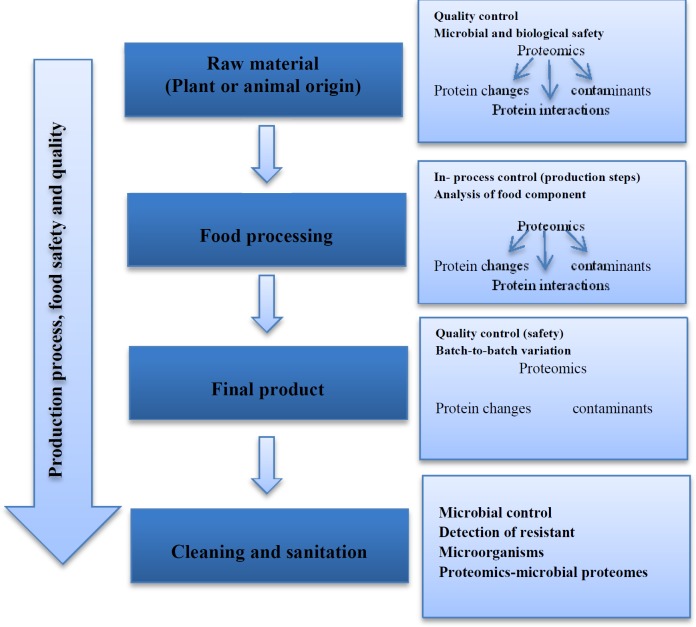
Use of proteomics in the development pathway for food production, and assessing food safety, originality and quality (Dajana et al., 2010)

 Food and human nutrition make an important bio-mixture; therefore their quality control and safety are very essential. Since proteins are the main constituents of foods, proteomic technology can monitor and characterize the protein content of foods and their changes during production using two-dimensional polyacrylamide gel electrophoresis (2D-PAGE) and chromatography techniques in combination with mass-spectrometry. Proteomics technology can be used for detection, validation, optimization, and also quality control of food industry (51). Concerning food science, food-proteomics methods can help to identify quality biomarkers to design better and safer foods. Therefore, proteomics may help food-producers to provide foods which give more guarantees of human health and safety. Thus, modern nutritional research focuses on health promotion, performance improvement, disease prevention, protection against toxicity, and stress. Some of the side effects of foods are due to contamination with microorganisms or their corresponding endo/exotoxins. Several numbers of human hospitalizations and even deaths happen because of microbial contaminations each year. Proteomics is a useful tool for identification of microbial contaminations and their toxins. In some cases, food products with animal origin such as seafood and milk create allergic reactions; in this regard, proteomics can be a very important tool for detection of allergens origin (52). Proteomic techniques are increasingly used for quality control and safety of raw materials in food industry as well. Most of the proteomic researches in the food technology are performed by the use of comparative 2D-PAGE and quantitative proteomics (53). Figure 5 shows proteomics tools in the processing of food production and quality controls (2). Proteomics applications can be divided into several groups in food investigation including cereal grains, fruits, eggs, meat products, and seafood. For example, nutritional and safety evaluations of transgenic rice conducted by Wang *et al.* in 2012 did not reveal significant differences between transgenic and non-transgenic rice. Because of a single gene insertion and environmental influences, approximately 20 proteins were differently modulated in the transgenic plants (54). The first comprehensive identification of rice grain proteins was conducted by Lee and Koh in 2011. In this study, with a label free shotgun proteomic approach, a large number of rice grain proteins were identified and the expression patterns of reproducibly identified proteins during rice grain development compared. The results of this study showed that proteins involved in glycolysis, tricarboxylic acid cycle, lipid metabolism, and proteolysis accumulated at higher levels in fully mature grain in comparison with grain developing stages, suggesting that the accumulation of these proteins during the desiccation stages may be associated with the preparation of proteins required for germination (55). In case of milk, Hinz *et al*., (2012) compared bovine, caprine, buffalo, equine, and camel milk protein profiles by 2D-PAGE, matrix assisted laser desorption ionization-time of flight mass spectrometry (MALDI-TOF/MS). The results of this study showed no beta-lactoglobulin protein present in the camel milk even though five isoforms of Kappa-casein protein were identified (56). Many studies have been performed in nutrition-proteomics (such as yogurt, egg, meat, etc.) field. Table 2 shows overview of some recent publications in the field of various food products.

 With rapidly growing global population, climate changes and increasing need for natural resources, food production with efficient crop yield is critical. To achieve these aims, novel methodology for vitality of seeds and protecting crops against stress are required. “Omics” technologies are promising tools for such objectives. A specific advantage of proteomics over other “Omics” techniques is the capacity to determine the functional impact of protein modifications on crop plant productivity (57). The application of proteomics for analyzing crop plants increased during the last decade. Proteomics studies have identified numerous proteins that play crucial roles in plant growth and development. Seeds are one of the most important factors in crop production, as crop yield is related to seed vitality. He and Yang (2013) applied proteomics to study the regulation of rice seed germination (58). One of the traditional breeding methods is transgenic technique to obtain crops with desired qualities (59). Evaluation of these genetically modified crops with proteomic methods is essential. Another aspect of proteomic studies in agriculture is related to interaction between crops and other organisms that influence the growth yield of crops (60). Proteomic analysis can complement molecular genetics approaches for studying the mechanisms by which pathogens attack cereal crops. Also proteomics approach is an efficient tool to analyze agriculture crop biomass (61). Systems biology analysis will also help the breeding of robust crop plants that are tolerant to environmental stresses and have high nutritional value (62).

**Table 2:** Some of recent reports related to the proteomics applications in field of nutrition

**Sample**	**Target**	**Methods**	**Results**	**Study**
Rice	Rice grain development	Label-free quantitative shotgun proteomics	4172 non-redundant protein identification high level accumulation of involving proteins in glycolysis, TCA cycle, lipid metabolism and proteolysis in mature grain compared to grain developing stage	Lee and Koh., 2011 (55)
Rice	Safety and quality assessment of transgenic rice	2DE, MALDI-TOF/TOF-MS/MS	21 proteins were up- or down-regulated as a consequence of environmental influence (WT01 vs. WT02) 20 to 22 protein levels were differentially modulated in transgenic rice seeds in comparison to their non-transgenic counterparts (T01 vs. WT01; T02 vs. WT02)	Wang *et al*., 2012 (54)
Milk	coagulation properties of bovin milk related to protein isoform, evaluation of PTMs	2DE, MALDI-TOF/MS	High prevalence of B variant of B-CN, K-CN and B-LG proteins in good coagulation milk Poorly coagulation milk associated with the B-CN variant A(2), K-CN variant A or E and B-LG variant A or C	Jensen *et al*., 2012 (83)
Milk	Comparison of bovine, caprine, buffalo, equine, and camel milk protein profiles	2DE, MALDI-TOF/MS	No B-lactoglobulin protein in camel milk Identification of five isoforms of K-casein protein in camel milk	Hinz *et al*., 2012 (56)
Milk	Assessment of protein oxidation during milk thermal processing	SDS-PAGE, MALDI-TOF/MS, Western blot	α-lactalbumin displayed enhanced oxidation compared to β-lactoglobulin despite its lower concentration in milk	Meyer *et al*., 2012 (84)
Milk	Peptide profile of cheese made from different types of milk	2DE, MALDI-TOF/MS, Q-TOF/MS, LC-ES/MS	identification of species-specific peptides the peptide profile of Teleme cheese is typical of other cheese	Pappa *et al*. 2008 (85)
Yogurt	functional peptides identification in yogurt	Solid-phase extraction, LC-TOF/MS	Significant number of peptides was reported as angiotensin converting enzyme (ACE) inhibitors and nine of them were antihypertensive	Kunda *et al*.,2012 (86)
Egg	Egg white protein characterization of different egg varieties (organic, omega 3 enriched)	2DE, LC-MS/MS	Levels of 19 protein were different and ovalbumin, cystatin, avidin and albumin precursor were not different among six egg varieties	Wang *et al*., 2012 (87)
Meat	Meat quality : protein quality markers related to oxidation processes	2DE, LC-MS/MS	five protein groups (alburedoxins, annexins, lipid transporters and enzymes of aerobic respiration), from which a link with lipid oxidation can be established	Sayd *et al*., 2012 (88)
Meat	Microbial safety of meat: staphylococcus enterotoxin in chicken	LC-MS, isotope labeling	Results showed that proteomics-based methods are effective for detect, confirm and quantify of SEB concentration in food metrices	Bao *et al*., 2012 (89)
Fish	Fish product safety: Bacterial identification	MALDI-TOF	Compilation of a spectral library including fingerprints and spectral data of the 20 Gram-positive bacterial species with the importance in seafood quality and safety	Böhme *et al*. 2011 (90)

## Conclusion

 Due to the growth of proteomics technology and unimaginable growth of scientific articles using proteomics approaches, proteomics applications in a wide range of investigations such as food industry, toxicology, development, neuroscience, and apoptosis would not be surprising. Food processing and its safety are important in the field of public health. Proteomics using different approaches has the power to characterize the proteinous components of foods and their changes during their production. Early diagnosis of diseases has been one of the main goals of physicians to choose a better and more effective treatment. Many studies have been carried out to determine the factors associated with different diseases as well as their molecular mechanisms. In such studies, the new proteomics technology has been of great value. In fact, one of the main objectives of proteomics is to identify and discover the disease-related biomarkers and drug targets. Because of many confronting factors, such as the complexity of the body fluids, and low abundance of a protein biomarker, there are many challenging steps for biomarker discovery to validate. Despite the existing challenges, there are other “omics” methodologies such as peptidomics, and metabolomics along with bioinformatics methods to help systemic study of disease for ideal biomarker introduction. In summary, a combined all of these “omics” approaches reveals new insight in various fields of proteomics research.
